# Salinity affects microbial function genes related to nutrient cycling in arid regions

**DOI:** 10.3389/fmicb.2024.1407760

**Published:** 2024-06-14

**Authors:** Yan Li, Wenjing Li, Lamei Jiang, Eryang Li, Xiaodong Yang, Jianjun Yang

**Affiliations:** ^1^Department of Ecology, College of Ecology and Environment, Xinjiang University, Urumqi, China; ^2^Key Laboratory of Oasis Ecology, Ministry of Education, Urumqi, China; ^3^Xinjiang Jinghe Observation and Research Station of Temperate Desert Ecosystem, Ministry of Education, Jinghe, China; ^4^Technology Innovation Center for Ecological Monitoring and Restoration of Desert-Oasis, Urumqi, China; ^5^College of Life Science, Xinjiang Agricultural University, Urumqi, China; ^6^Department of Geography and Spatial Information Technology, Ningbo University, Ningbo, China

**Keywords:** salinity, carbon cycle, nitrogen cycle, phosphorus cycle, sulfur cycle, arid desert areas

## Abstract

**Introduction:**

Salinization damages soil system health and influences microbial communities structure and function. The response of microbial functions involved in the nutrient cycle to soil salinization is a valuable scientific question. However, our knowledge of the microbial metabolism functions in salinized soil and their response to salinity in arid desert environments is inadequate.

**Methods:**

Here, we applied metagenomics technology to investigate the response of microbial carbon (C), nitrogen (N), phosphorus (P), and sulfur (S) cycling and the key genes to salinity, and discuss the effects of edaphic variables on microbial functions.

**Results:**

We found that carbon fixation dominated the carbon cycle. Nitrogen fixation, denitrification, assimilatory nitrate reduction (ANRA), and nitrogen degradation were commonly identified as the most abundant processes in the nitrogen cycle. Organic phosphorus dissolution and phosphorus absorption/transport were the most enriched P metabolic functions, while sulfur metabolism was dominated by assimilatory sulfate reduction (ASR), organic sulfur transformation, and linkages between inorganic and organic sulfur transformation. Increasing salinity inhibited carbon degradation, nitrogen fixation, nitrogen degradation, anammox, ANRA, phosphorus absorption and transport, and the majority of processes in sulfur metabolism. However, some of the metabolic pathway and key genes showed a positive response to salinization, such as carbon fixation (*facA*, *pccA*, *korAB*), denitrification (*narG*, *nirK*, *norBC*, *nosZ*), ANRA (*nasA*, *nirA*), and organic phosphorus dissolution processes (*pstABCS*, *phnCD*, *ugpAB*). High salinity reduced the network complexity in the soil communities. Even so, the saline microbial community presented highly cooperative interactions. The soil water content had significantly correlations with C metabolic genes. The SOC, N, and P contents were significantly correlated with C, N, P, and S network complexity and functional genes. AP, NH4+, and NO3− directly promote carbon fixation, denitrification, nitrogen degradation, organic P solubilization and mineralization, P uptake and transport, ASR, and organic sulfur transformation processes.

**Conclusion:**

Soil salinity in arid region inhibited multiple metabolic functions, but prompted the function of carbon fixation, denitrification, ANRA, and organic phosphorus dissolution. Soil salinity was the most important factor driving microbial functions, and nutrient availability also played important roles in regulating nutrient cycling.

## 1 Introduction

Microorganisms are an important part of soil ecosystems and play a crucial role in the health of the ecosystem (Che et al., 2019). Salinization is a global problem that affects element turnover and soil quality. It has been reported that more than 900 million hectares of soil worldwide is subject to different degrees of salt stress (An et al., 2023). Soil salinization inhibits microbial growth and respiration (Rath et al., 2017), and affects the biological distribution, diversity and structure of soil microbial communities in multiple ecosystems (Gavrichkova et al., 2018; Rath et al., 2019; Zhang et al., 2019; Zhao et al., 2020). Global studies on saline–alkali land have shown that salinization not only affects microbial community composition—e.g., halophiles (salt-tolerant microbes) may thrive under saline conditions, while sensitive species are suppressed or eliminated (Boujelben et al., 2012; Yang et al., 2022)—but also the altered microbial activities can impact microbiomes’ functional genes, such as genes associated with carbon degradation and nitrogen cycling (Zhang G. L. et al., 2021) or nutrient cycling (Widdig et al., 2020; Zhang X. Y. et al., 2021; Liang et al., 2022).

There are also inconsistencies in the effects of salinity on soil microbial communities. For example, microbial diversity has shown positive (Marinari et al., 2012), negative (Zhao et al., 2020), and independent (Zhang X. Y. et al., 2021) relationships with increasing salinity. It has been reported that increased soil salinity decreased the carbohydrate metabolism and gene abundances of glycosyl transferases and glycoside hydrolases. In contrast, the enzyme-active genes of carbohydrate esterases, along with their auxiliary activities, were positively associated with soil salinity (Yang et al., 2022). Salt significantly inhibited the carbohydrate metabolism function and expression of methane-metabolism-regulatory genes in saline–alkaline soil (Yang et al., 2023). However, one study in QTP showed that salinity promotes functional genes associated with carbon degradation, denitrification, and sulfur oxidation (Li Y. Q. et al., 2021). These contradictory results may be due to the differences in climatic conditions, ecosystems, or salt concentrations between studies.

Other than salinity, climatic conditions (such as temperature and precipitation) and edaphic variables (e.g., pH and nutrient availability) also play important roles in the distribution of microbial communities’ functions. The main factors driving the functions of microbial communities are still controversial in different ecosystems or soil conditions. For instance, the bacterial community composition and diversity were significantly impacted by the soil pH, electrical conductivity, Na^+^, K^+^, Cl^−^, and CO_3_^2−^ in saline soils of Northeast China (Liang et al., 2022), while the key driving factors of microorganisms were oxygen and pH (Hollister et al., 2010). Therefore, more efforts are needed to reveal the specific driving factors shaping microbial communities’ structure and function in diverse ecosystems.

Desert ecosystems are characterized by an arid climate, low precipitation, high evaporation, and sparse vegetation. Despite their harsh environmental conditions, deserts are home to a variety of plants and animals that have adapted to survive in these challenging environments. They can provide important ecosystem services, such as soil stabilization, nutrient cycling, and habitats for wildlife, and play a crucial role in maintaining global biodiversity and supporting local communities that rely on desert resources for food and livelihoods (Whitford, 2002). Due to the distinctiveness of the climate in arid areas where precipitation is low but the evaporation is very strong, soil salinization, especially in large basin areas, becomes a serious ecological problem that negatively impacts land-use efficiency and the provision of ecosystem services. Microbial communities in natural saline soil not only can be used as a model system to explore the relationship between soil diversity and activities, but also represent the potential gene reserves of biotechnology applications for improving and protecting the soil quality (Canfora et al., 2014). In addition, microbiomes have potential for biotechnological applications in the improvement and conservation of saline soils (Canfora et al., 2015). However, the research on the diversity, composition, and driving factors of functional genes in arid saline soils is relatively lacking. Therefore, extensive research is needed to investigate the structure and function of organisms in natural saline–alkali soils in arid regions.

Previously, the microbial community diversity and structure of saline soils in arid desert areas of Xinjiang were revealed (Yang et al., 2022). In the present study, we used metagenomic sequencing data to investigate the microbiome functions in salinized arid regions, in an effort to elucidate (1) the effects of salinization on microbial carbon (C), nitrogen (N), phosphorus (P), and sulfur (S) metabolism processes, along with the related key functional genes; (2) the response of microbial networks to salinity; and (3) the primary edaphic variables influencing microbial metabolic functions.

## 2 Materials and methods

### 2.1 Field investigation and saline soil collection

We randomly selected 12 sites across Xinjiang, and a total of 36 saline samples were collected on Sep 2021. The detailed information of the sampling sites were listed in Supplementary Figure S1 and Supplementary Table S1. From each site, three samples were collected from the 0–20 cm surface layer and packed in a 50 mL sterile tube after the removal of any stones or plant debris. The samples were temporarily kept at −10°C in a car-carried refrigerator during transportation. After they were returned to the laboratory, each of the samples was divided into two parts, of which one part was air-dried at room temperature for physicochemical property tests after sieving through a 2 mm filter, while the other was kept at −80°C for genomic DNA extraction.

### 2.2 Determination of soil properties

The soil moisture was calculated from the weight difference between soil weighed before and after drying at 105°C for 48 h. The soil pH was determined using an electrode pH meter (DDSJ-319 L, Shanghai, China) in a 1:2.5 soil/water (w/v) suspension. The total organic carbon (TOC) was estimated using a UV–visible spectrophotometer (UV-1200, Shanghai, China) after the soil samples were oxidized with K_2_Cr2O4. The concentrations of total phosphorus (TP) and available phosphorus (AP) were determined by Mo-Sb colorimetric analysis using the UV-1200 spectrophotometer after the soil was digested with a HClO_4_-H_2_SO_4_ solution for 60 min. The total nitrogen (TN) was analyzed using an AA3 flow analyzer (SEAL Analytical GmbH, Norderstedt, Germany) after digestion with concentrated sulfuric acid and perchloric acid. The nitrate–nitrogen ( ${\mathrm{NO}}_{3}^{-}$ -N) and ammonium–nitrogen ( ${\mathrm{NH}}_{4}^{+}$ -N) were determined using the AA3 flow analyzer after extraction in 1 mol/L KCl.

### 2.3 Metagenomic sequencing, assembly and function annotation

The total DNA was extracted from each soil sample using a FastDNA SPIN Kit for Soil (MP Biomedicals, Cleveland, United States). The quantity and quality of the isolated DNA were evaluated using a NanoDrop 2000 spectrophotometer (Thermo Fisher Scientific, Waltham, MA, United States) and 1% agarose gel electrophoresis, respectively. The genomic DNA (~1 ug) was randomly sheared into short fragments of about 350 bp using a Covaris M220 (Gene Company Limited, China). The obtained fragments were end-repaired, A-tailed, and ligated with an Illumina adapter and unique barcode, PCR-amplified, size selected, and purified to prepare paired-end library using an Ultra DNA Library Prep Kit for Illumina (New England Biolabs, United States). A NanoDrop 2000 was used to quantify the constructed library, and a Agilent 2100 Bioanalyzer was used to ascertain the insert size of the library. The quantified libraries were pooled and sequenced using the Illumina HiSeq PE500 platform at Novogene Bioinformatics Technology Co., Ltd. (Tianjin, China).

The raw sequencing data were filtered with Readfq to obtain clean data after removing reads with low-quality bases (quality threshold <38) exceeding 40 bp in length, reads with N bases reaching 10 bp, and reads overlapping with adapters of length > 15 bp. MEGAHIT software (v1.0.4-beta) was used to assemble the clean reads to scaffolds. The assembled scaffolds were interrupted at the N connection to obtained scaftigs (continuous sequences without N within scaffolds) (Mende et al., 2012; Nielsen et al., 2014). MetaGeneMark (V3.05) was used to perform gene predictions for scaftigs (≥500 bp) with the default parameters (Chen and Pachter, 2005; Li and Godzik, 2006; Zhu et al., 2010), and the predicated genes less than 100 nt were discarded. The CD-HIT software V4.5.8 was used to eliminate redundancies and obtain the non-redundant initial gene catalog (Fu et al., 2012). Then, the clean reads were mapped to the initial gene catalog using Bowtie2 (Langmead and Salzberg, 2012), with ≥95% identity, and the gene abundances were evaluated in each sample. Genes with total reads ≤2 in each sample were removed to acquire the final unigenes (Li et al., 2014). DIAMOND V0.9.9 (Buchfink et al., 2015) was used for alignment of unigenes with those of bacteria, fungi, archaea, and viruses extracted from NCBI’s NR database (Version 2018-01-02), with a cutoff *e*-value of 10^−5^. The LCA algorithm was adopted to determine the species annotation information using MEGAN (Huson et al., 2011). The functional genes related to microbial carbon (C), nitrogen (N), phosphorus (P), and sulfur (S) metabolism were screened from the KEGG databases using the KEGG Orthology-Based Annotation System (KOBAS).

### 2.4 Statistical analyses

We evaluated the functional similarity of microbial carbon (C), nitrogen (N), phosphorus (P), and sulfur (S) metabolic function between the two saline groups via principal coordinates analysis (PCoA) based on the Bray–Curtis distance within the *vegan* package. The *t*-test was used to analyze the differences in the relative abundance of functional genes involved in C, N, P, and S turnover between the hypersaline and saline soils. The false discovery rate (FDR) was used to correct the *p*-values of multiple correlations in order to reduce the number of false positive results (Benjamini et al., 2006). The network was constructed with differentially expressed functional genes between the two saline groups. The level interaction between functional genes and environmental factors was calculated using Spearman’s correlation coefficient. The functional genes and environmental factors with significant correlations (FDR < 0.001) were used to construct the interaction network for the microbial C, N, P, and S nutrition cycles. All networks were visualized using the interactive platform CYTOSCAPE 3.6.1. In the network, nodes represent nutritional circulation function genes or environmental factors, while the edge connecting two nodes represents the positive and negative correlation between them. Structural equation modeling (SEM) was used to analyze the relative contributions of soil properties (AP, ${\mathrm{NH}}_{4}^{+}$ , ${\mathrm{NO}}_{3}^{-}$ , SWC, pH, TP, EC) to the C, N, P, and S metabolic functions, implemented in Amos 24.0. Before calculating the SEM, the correlation analysis was used to reduce the number of variables and screen the factors influencing the nutrient cycling (Supplementary Table S3). The EC, SWC, TP, AP, ${\mathrm{NH}}_{4}^{+}$ , and ${\mathrm{NO}}_{3}^{-}$ were retained after discarding of variable with coefficient r > 0.8. To further reduce the variables, the principal component analysis (PCA) was used to simplify the variables to three principal components, PC1 mainly composed of AP, ${\mathrm{NH}}_{4}^{+}$ , and ${\mathrm{NO}}_{3}^{-}$ (soil PC1), PC2 included SWC and TP (soil PC2), and PC3 consisted of EC (soil PC3).

## 3 Results

### 3.1 Soil characteristics

The EC values determined for the saline soil ranged from 12.87 mS·cm^−1^ to 110.9 mS·cm^−1^; the pH values ranged from 7.59 to 9.76 (Supplementary Table S1). We previously divided soil samples into two groups—saline (average EC < 30 mS·cm^−1^) and hypersaline (average EC ≥ 30 mS·cm^−1^)—according to the USDA soil taxonomy criteria (Yang et al., 2022). Therefore, the present study adopted the same classification, in order to provide insights into the effects of salinization on microbial functional genes participating in the C, N, P, and S metabolism.

### 3.2 Microbial functions and related genes

**Figure 1:**
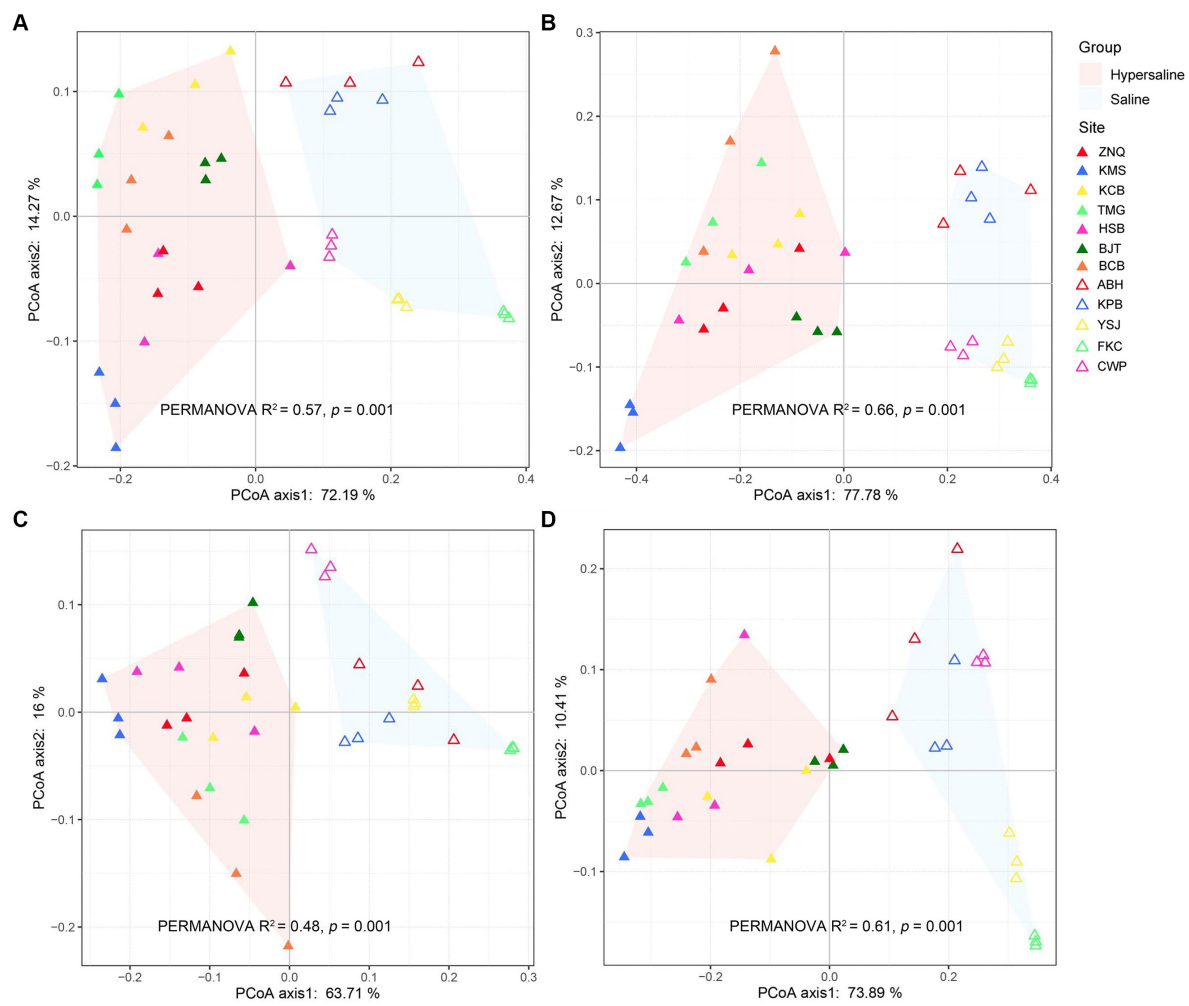
PCoA plot constructed with carbon **(A)**, nitrogen **(B)**, phosphorus **(C)**, and sulfur **(D)** metabolic gene dataset indicating that the functional composition of microbial communities was separated along axis 1, corresponding to the saline and hypersaline groups.

PCoA revealed that the functional gene composition was clearly separated along PC1, which explained 72.9, 77.78, 63.71, and 73.89% of the total variation in the metabolism of C, N, P, and S, respectively (Figure 1). This indicated that the salt concentration had a strong effect in altering the functions of soil microbiomes, which is consistent with a previous report of significant differences in community structure (Yang et al., 2022). PERMANOVA also showed significant differences between the saline and hypersaline groups in terms of functional genes involved in C, N, P, and S turnover processes (*R*^2^ = 0.57, 0.66, 0.48, and 0.61, respectively; all *p* < 0.01) (Figure 1).

#### 3.2.1 Carbon cycle processes and key genes

**Figure 2:**
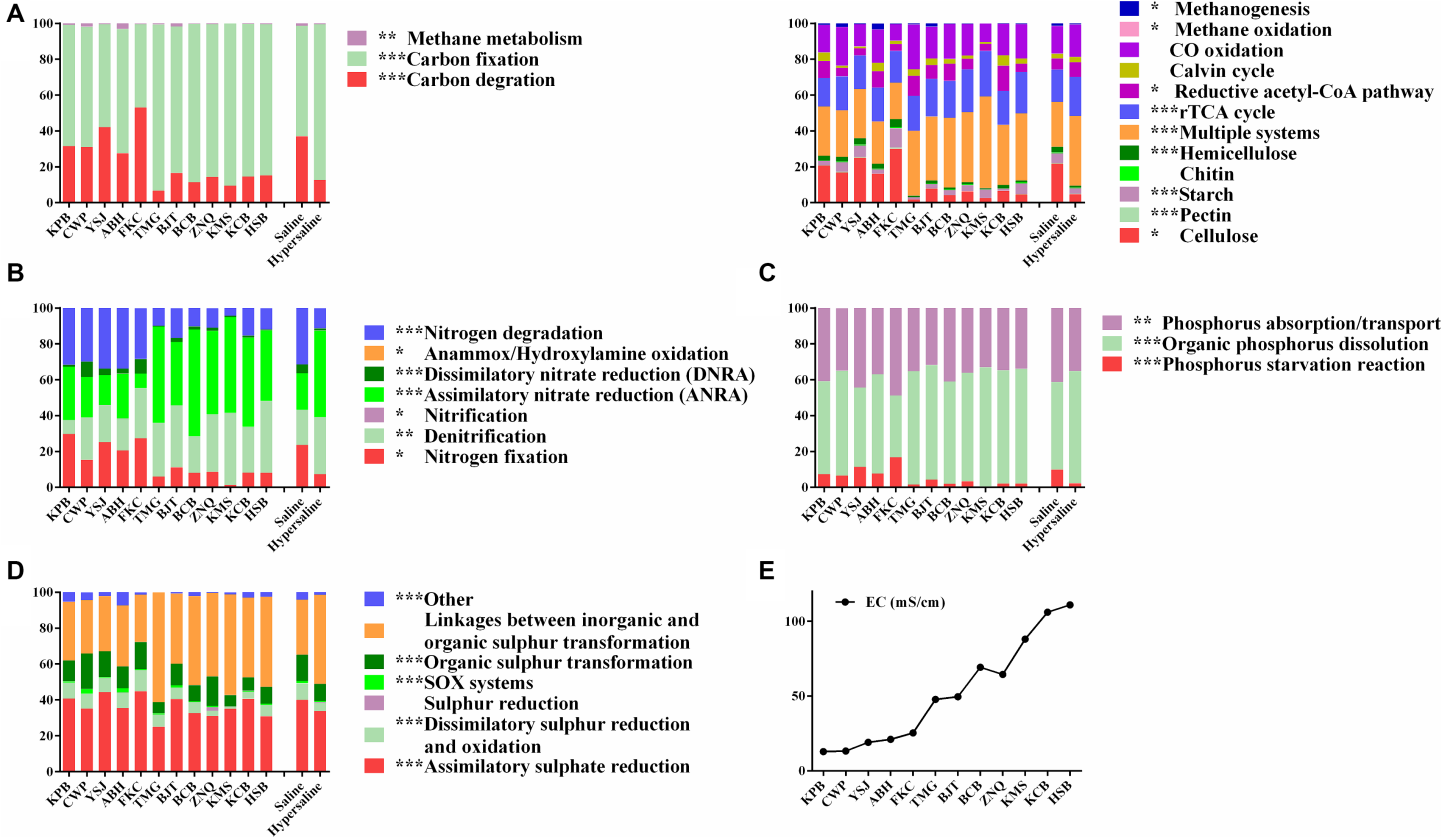
The relative abundance of carbon **(A)**, nitrogen **(B)**, phosphorus **(C)**, and sulfur **(D)** metabolic processes at each site, and statistical analysis in the saline and hypersaline soil groups; E, plot of EC values at each site; *, **, and *** indicate significant differences between the saline and hypersaline groups at FDR < 0.05, FDR < 0.01, and FDR < 0.001, respectively.

All functional genes involved in the carbon cycle were divided into carbon degradation, carbon fixation, and methane metabolism (Supplementary Table S2). Carbon fixation genes showed the highest standardized abundance, followed by C degradation genes. The abundance of carbon degradation and methane metabolism in saline soils was significantly more prominent than that in hypersaline environments (FDR < 0.01), whereas the carbon fixation process in hypersaline environments was enriched, significantly higher than that in saline soils (FDR < 0.01) (Figure 2A). For carbon fixation, the relative abundance of the reductive tricarboxylic acid cycle (rTCA cycle) and reductive acetyl-CoA pathway, showed high abundance in the high-salt environment (FDR < 0.05). For carbon degradation and methane metabolism, the abundance of functional genes related to starch, pectin, cellulose, hemicellulose, methane oxidation, and methanogenesis in saline soils was significantly higher than that in hypersaline soils (FDR < 0.05) (Figure 2A).

**Figure 3:**
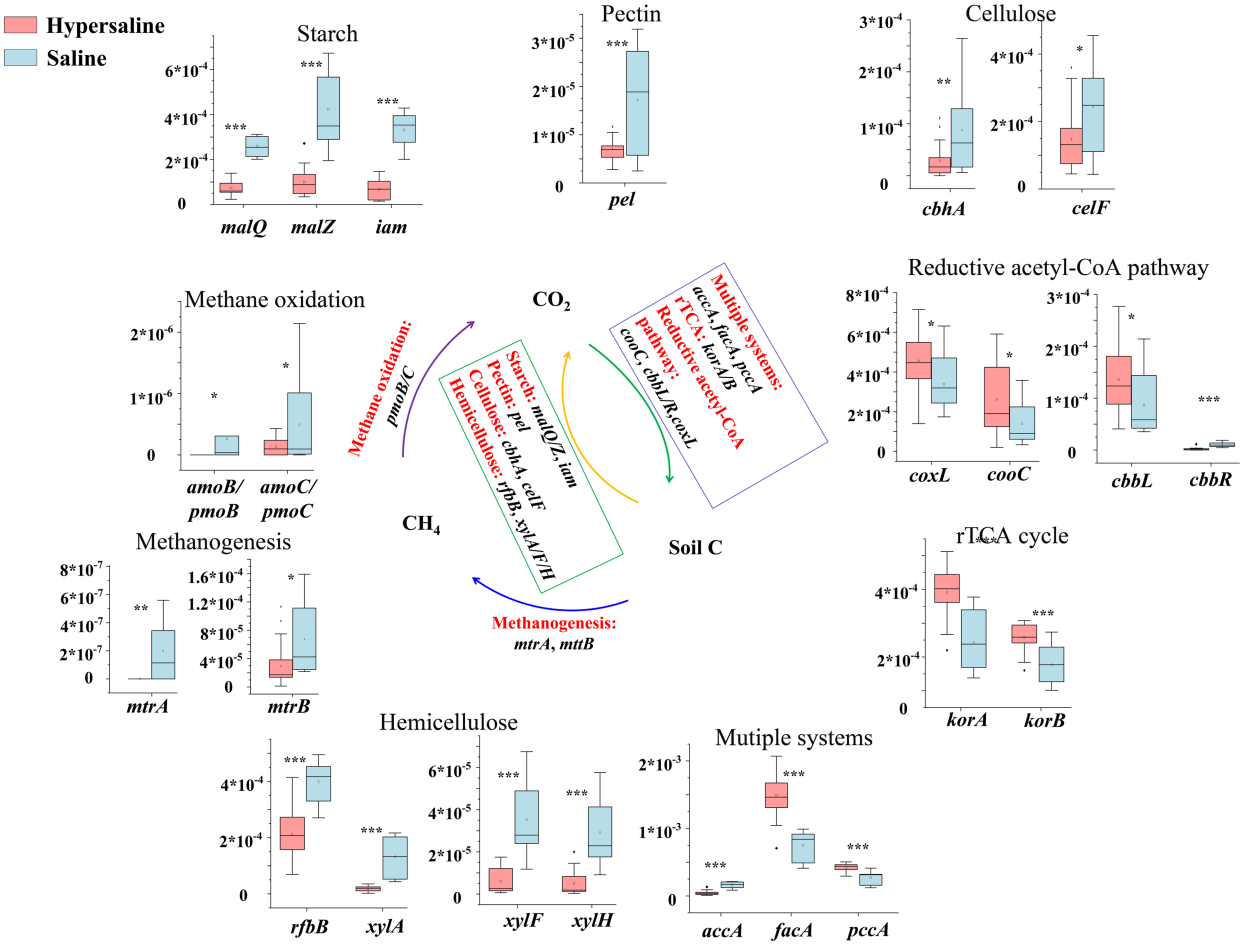
Diagram depicting the carbon cycling processes; the middle carbon cycling diagram is adapted with permission from Hu et al. (2022), licensed under Elsevier. *, **, and *** indicate significant differences in functional genes between hypersaline and saline soils at FDR < 0.05, FDR < 0.01, and FDR < 0.001, respectively.

The genes involved in the rTCA cycle (*korA* and *korB*), the multiple systems (*pccA* and *facA*), and the reductive acetyl-CoA pathway (*coxL*, *cooC*, and *cbbL*) were more abundant in hypersaline soils than in saline soils (*p* < 0.05), indicating that the carbon fixation potential in hypersaline soils was higher (Figure 3). For the carbon degradation, the genes related to starch degradation (*malQ*, *malZ*, and *iam*), pectin degradation (*pel*), cellulose degradation (*cbhA*, *celF*), and hemicellulose degradation (*rfbB*, *xylA*, *xylF*, and *xylH*) were significantly higher in saline soils than those in hypersaline soil (*p* < 0.05). This suggests that high salinity environment (hypersaline) had lower carbon degradation potential. Besides, the relative abundance of methane-oxidation- and methanogenesis-related genes was significantly higher in saline plots than in hypersaline plots (*p* < 0.05) (Figure 3).

#### 3.2.2 Nitrogen cycle processes and key genes

Assimilatory nitrate reduction (36.70%), denitrification (26.66%), nitrogen degradation (19.72%), and nitrogen fixation (14.17%) were the main pathway in the nitrogen cycle. Among these, the dissimilatory nitrate reduction, nitrification, nitrogen fixation, anammox/hydroxylamine oxidation, and nitrogen degradation in saline soils were significantly higher than those in hypersaline soils (FDR < 0.05). However, denitrification and assimilatory nitrate reduction were significantly higher in hypersaline plots than in saline plots (FDR < 0.01) (Figure 2B).

**Figure 4:**
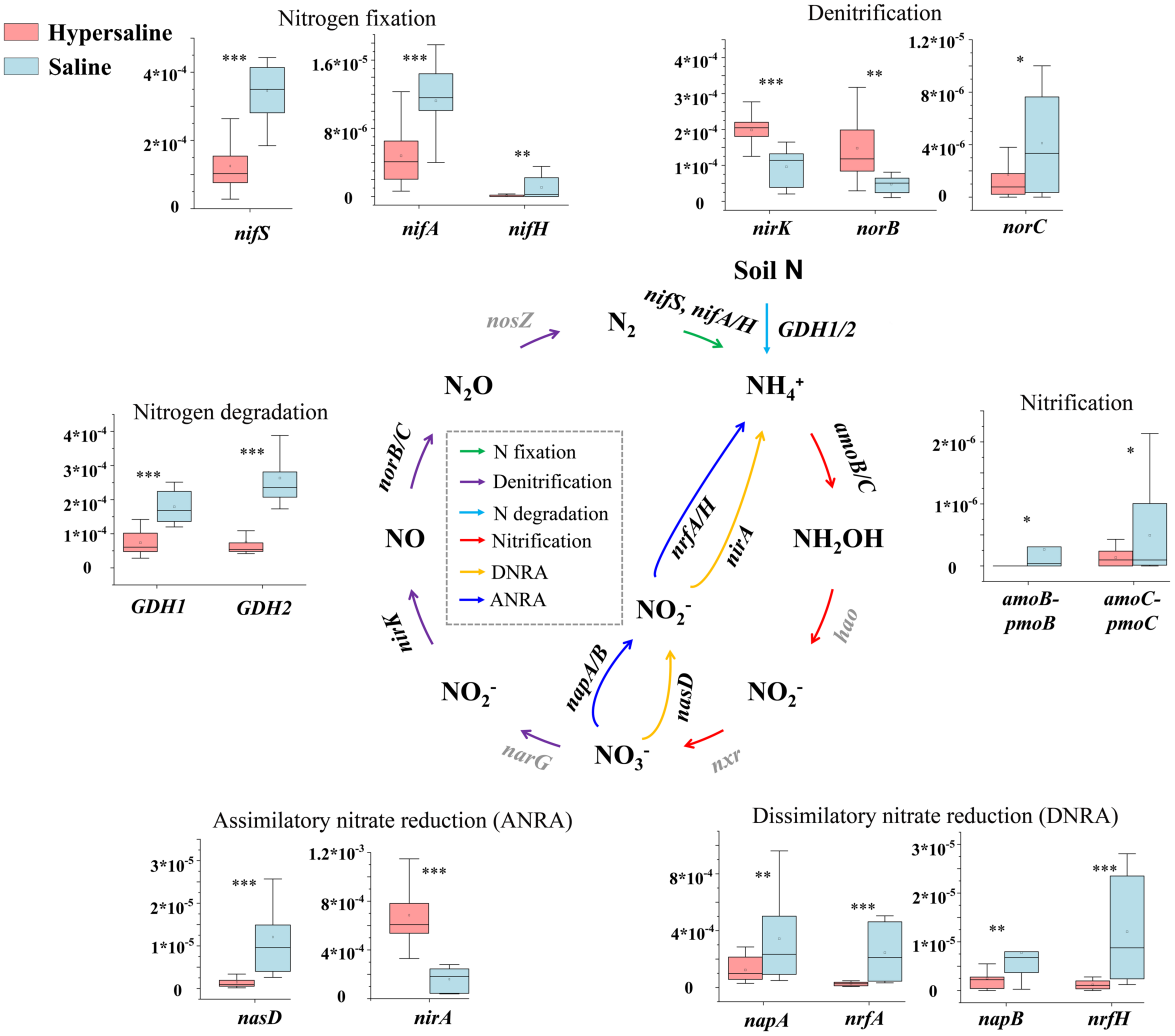
Diagram depicting the nitrogen cycling processes. The middle nitrogen cycling diagram is adapted with permission from Hu et al. (2022), licensed under Elsevier. *, **, and *** indicate significant differences in functional genes between hypersaline and saline soils at FDR < 0.05, FDR < 0.01, and FDR < 0.001, respectively.

The functional genes *amoB*, *amoC* ( ${\mathrm{NH}}_{4}^{+}$ to NH_2_OH), *nifS*, *nifA*, *nifH* (N_2_ to ${\mathrm{NH}}_{4}^{+}$ ), *norC* ( ${\mathrm{NO}}_{2}^{-}$ to ${\mathrm{NO}}_{3}^{-}$ ), *nrfA*, *nrfH* ( ${\mathrm{NO}}_{2}^{-}$ converted to ${\mathrm{NH}}_{4}^{+}$ ), *nasD*, *napA*, *napB* ( ${\mathrm{NO}}_{3}^{-}$ converted to ${\mathrm{NO}}_{2}^{-}$ ), and *norC* (NO converted to N_2_O) were significantly more abundant in saline soils than in hypersaline soils (FDR < 0.05). However, it is worth noting that the functional genes *nirK* and *norB*, which regulate the conversion of ${\mathrm{NO}}_{2}^{-}$ to NO and NO to N_2_O, respectively, were significantly more abundant in hypersaline soils than in saline soils (FDR < 0.05). In addition, *nirA*, which converts ${\mathrm{NO}}_{2}^{-}$ to ${\mathrm{NH}}_{4}^{+}$ , also had a high abundance level in the hypersaline soils (FDR < 0.05) (Figure 4).

#### 3.2.3 Phosphorus cycle processes and key genes

Phosphorus absorption/transport and organic phosphorus dissolution were the dominant pathway identified in the phosphorus (P) cycle, accounting for an average of 56.89 and 37.63% of the total abundance, respectively, while phosphorus starvation reactions accounted for 5.48% of the P cycle (Figure 2C). In saline soils, phosphorus starvation reactions and phosphorus absorption/transport were significantly more prominent than in hypersaline soils, while organic phosphorus dissolution showed the opposite trend (Figure 2C).

**Figure 5:**
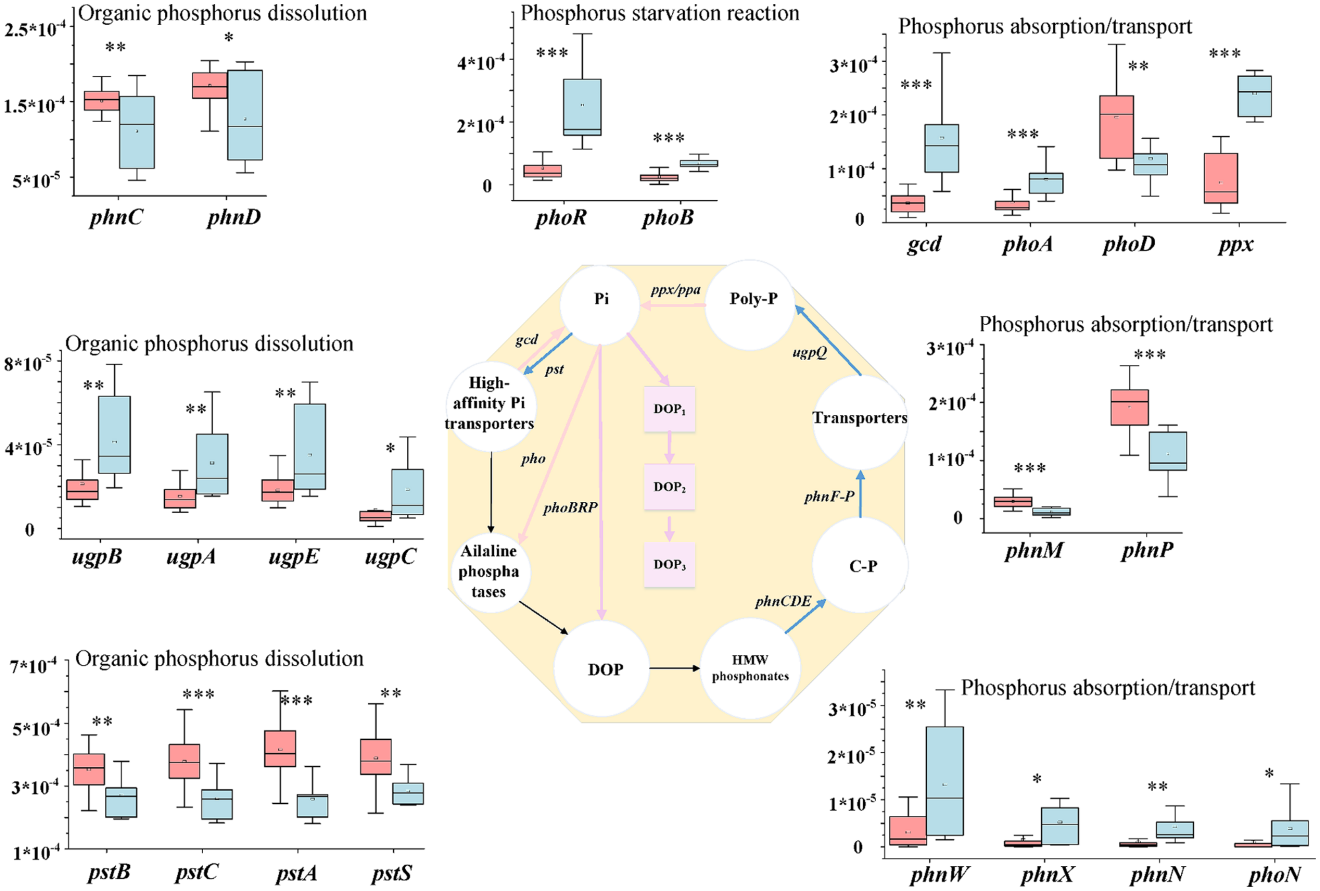
Diagram depicting the phosphorus cycling processes; the middle phosphorus cycling diagram is reprinted with permission from Li et al. (2023b), licensed under Elsevier. *, **, and *** indicate significant differences in functional genes between hypersaline and saline soils at FDR < 0.05, FDR < 0.01, and FDR < 0.001, respectively.

Among the genes involved in phosphorus starvation, *phoR* and *phoB* were significantly more abundant in saline soils than in hypersaline soils. The abundance of *phoR* in saline soils was 4.79 times that in hypersaline soils. Among the functional genes involved in inorganic phosphorus dissolution and organic phosphorus mineralization, the relative abundance of *ugpA*, *ugpB*, *ugpC*, and *ugpE* in saline soils was significantly higher than in hypersaline soils; in contrast, the genes *pstA,B,C,S* and *phnC,D* in high-efficiency phosphorus absorption systems had higher abundance in hypersaline soils than in saline soils (FDR < 0.05). In the process of phosphorus absorption and transportation, *phnW,X,N,M,P*, *phoA*, *ppx*, and *gcd* had higher abundance in saline soils than in hypersaline soils (FDR < 0.05), while the functional genes *phoD* and *phnP* showed the opposite trend (Figure 5).

#### 3.2.4 Sulfur cycle processes and key genes

**Figure 6:**
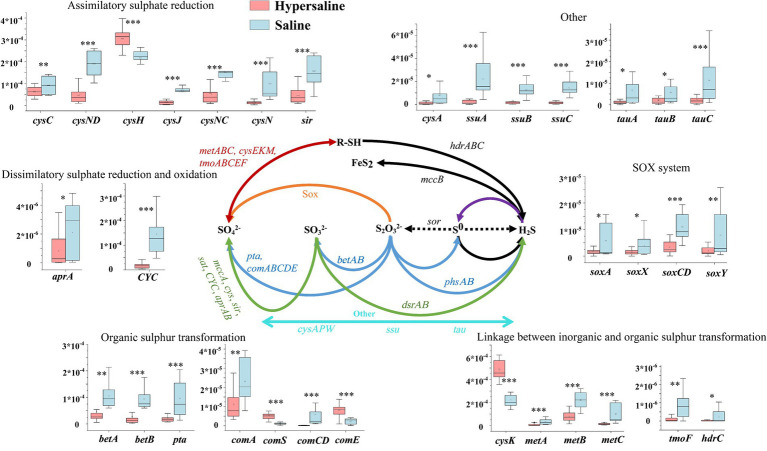
Diagram depicting the sulfur cycling processes. The middle sulfur cycling diagram is reprinted with permission from Li et al. (2022), licensed under John Wiley & Sons. *, **, and *** indicate significant differences in functional genes between hypersaline and saline soils at FDR < 0.05, FDR < 0.01, and FDR < 0.001, respectively.

In the sulfur (S) cycle, assimilative sulfur reduction (36.34%), organic sulfur transformation (11.67%), and linkages between inorganic and organic sulfur transformation (41.72%) were the main processes, accounting for 89.73% of the total S metabolism functions. The relative abundance of assimilatory sulfur reduction, organic sulfur transformation, dissimilar sulfur reduction and oxidation, and *SOX* systems in saline soils was higher than in hypersaline soils (FDR < 0.05) (Figure 2D). Among these, the relative abundance of *SOX* processes in hypersaline soils was 2.34–3.81 times that in saline soils. All of the S cycling genes declined in abundance with increasing salinity except for *cysK*, which was involved in linkages between inorganic and organic sulfur transformation, *comS* and *comE*, involved in organic sulfur transformation, and *cysH*, involved in assimilatory sulfur reduction processes (FDR < 0.05, Figure 6).

### 3.3 Network analysis of microbial functional genes

**Figure 7:**
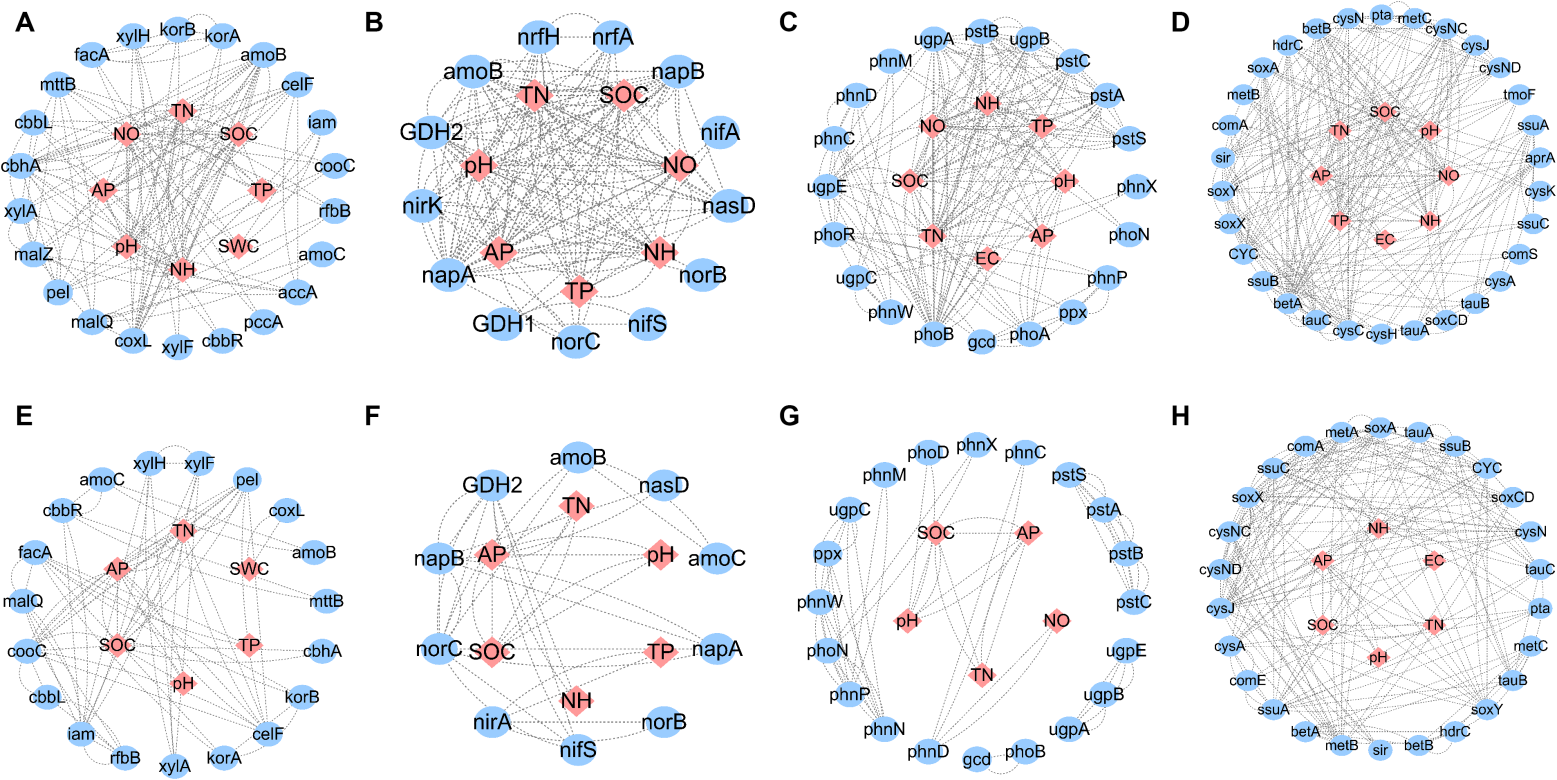
Network of functional genes and relationships with soil variables for carbon, nitrogen, phosphorus, or sulfur cycles in saline and hypersaline soils. **(A–D)** Indicate the carbon, nitrogen, phosphorus, or sulfur cycle in saline soils, respectively, while **(E–H)** indicate the carbon, nitrogen, phosphorus, or sulfur cycle in hypersaline soils, respectively. SWC, soil water content; SOC, soil organic carbon content; TN, soil total nitrogen content; TP, soil total phosphorous content; AP, soil available phosphorous content; NO, soil nitrate nitrogen; NH, soil ammonium nitrogen. Different lowercase letters represent significant differences between saline and hypersaline soils at *p* < 0.05.

The average shortest network parameter path length was 1.494–3.769, and the degree was 2.240–13.714. Most of the nodes in these networks were connected by several paths. The numbers of total links, nodes, positive links, degrees, neighborhood connectivity, and directed edges in saline soils were higher than those in hypersaline soils in all cycles: carbon (C), nitrogen (N), phosphorus (P), and sulfur (S). The positive chain ratio in saline environments was higher than that in hypersaline soils. The positive link ratios of the C cycle in hypersaline and saline soils were 0.811 and 0.882, respectively, while the ratios for the P cycle were 0.857 and 0.968, respectively. However, the positive chain ratios of the N and S cycles in hypersaline samples were higher than in saline samples. The S cycle network had the highest complexity (Figure 7; Table 1).

**Table 1:** Topological properties of the network for carbon, nitrogen, phosphorus, and sulfur cycling.

Topological properties	Carbon		Nitrogen		Phosphorus		Sulfur	
	Hypersaline	Saline	Hypersaline	Saline	Hypersaline	Saline	Hypersaline	Saline
Closeness centrality	0.314	0.334	0.609	0.676	0.752	0.384	0.604	0.343
Clustering coefficient	0.404	0.434	0.344	0.670	0.453	0.603	0.749	0.634
Degree	5.481	8.500	4.000	13.714	2.240	6.714	5.625	5.947
Neighborhood connectivity	3.556	5.601	2.991	8.942	2.450	8.083	6.347	7.006
Number of directed edges	5.484	8.500	4.000	13.714	2.240	6.714	5.625	5.947
Average shortest path length	3.769	3.131	1.859	1.546	1.494	2.775	1.793	3.048
Betweenness centrality	0.120	0.071	0.159	0.079	0.132	0.068	0.070	0.057
Topological coefficient	0.429	0.441	0.396	0.535	0.541	0.514	0.697	0.500
Positive links	60	120	36	142	48	182	174	200
Positive links/total links	0.811	0.882	1.000	0.986	0.857	0.968	0.967	0.885
Nodes	25	30	16	20	25	28	32	38

**Figure 8:**
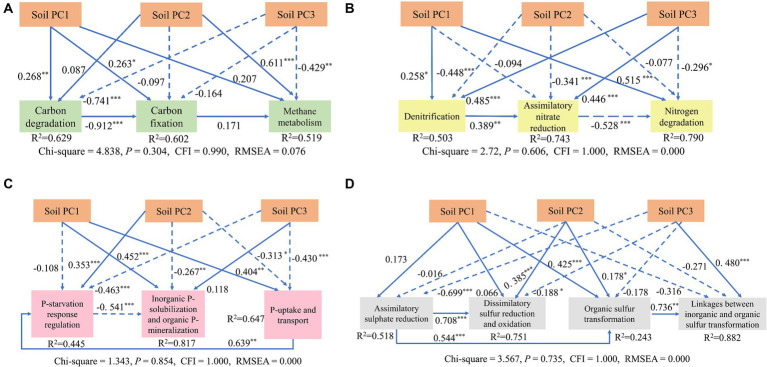
Results of a structural equation model (SEM) for determining how soil variables affected the carbon **(A)**, nitrogen **(B)**, phosphorus **(C)**, and sulfur **(D)** metabolism processes. Continuous and dashed arrows represent significant and nonsignificant relationships, respectively. Numbers adjacent to arrows are standardized path coefficients indicating the effect size of the relationship. Soil PC1 was composed of AP, ${\mathrm{NH}}_{4}^{+}$ , and ${\mathrm{NO}}_{3}^{-}$ ; Soil PC2 included SWC and pH; Soil PC3 included EC. The level of significance was *p < 0.05, **p < 0.01, and ***p < 0.001.

The functional genes and environmental factors in the network were screened out by sorting the values of the centrality of network parameters, and the factors that played a key role in the network were analyzed. The SOC, AP, N, SWC, and pH were key factors associated with microbial metabolic functional genes (Figure 8).

### 3.4 Effects of soil variables on microbial functions

SEM showed that soil PC3 (EC) had significant direct negative effects on carbon degradation and methane metabolism (path coefficient = −0.741*** and −0.429**, respectively); while soil PC2 (SWC and TP) had a significant positive direct impact on methane metabolism (path coefficient = 0.611***), and carbon degradation had a significant negative direct impact on carbon fixation (path coefficient = −0.912***) in the carbon cycle (Figure 8A). For the nitrogen cycle, PC3 (EC) had a significant positive direct effect on denitrification and assimilatory nitrate reduction (path coefficient = 0.485***, 0.446***), while it had a significant negative direct effect on nitrogen degradation (path coefficient −0.296*). Soil PC2 had a significant negative direct effect on denitrification (path coefficient = −0.448***) (Figure 8B). For the phosphorus cycle, PC3 (EC) had a significant direct negative impact on P starvation response regulation, P uptake/transport (path coefficient = −0.445**and −0.430**, respectively). Soil PC1 (AP, ${\mathrm{NH}}_{4}^{+}$ , and ${\mathrm{NO}}_{3}^{-}$ ) had a significant, positive, direct impact on organic P solubilization and mineralization, P uptake/transport (path coefficient = 0.452** and 0.404***, respectively), and soil PC2 had a significant positive direct impact on P starvation response regulation (path coefficient = 0.353***) (Figure 8C). For the sulfur cycle, PC3 (EC) had a significant positive direct influence on linkages between organic and organic sulfur transformation (path coefficient = 0.480***), while it had a negative effect on assimilatory sulfur reduction (path coefficient = −0.699***) (Figure 8D).

## 4 Discussion

Soil salinity regulate the microbial community structure, function and interaction networks. The functional gene diversity and composition could provide a deeper understanding of soil salinization on nutrient cycling in diverse ecosystems (Ma and Gong, 2013). In the present study, we investigated the effects of salinity on carbon (C), nitrogen (N), phosphorus (P), and sulfur (S) cycling and networks in soil samples collected from different saline–alkali environments. Finally, the key environmental factors influencing these functions were explored using structural equation models. Our results indicate that with increasing salinity, significant changes occur in microbial functions involved in C, N, P, and S metabolism.

### 4.1 Salinity alters microbial metabolic functions

**Figure 9:**
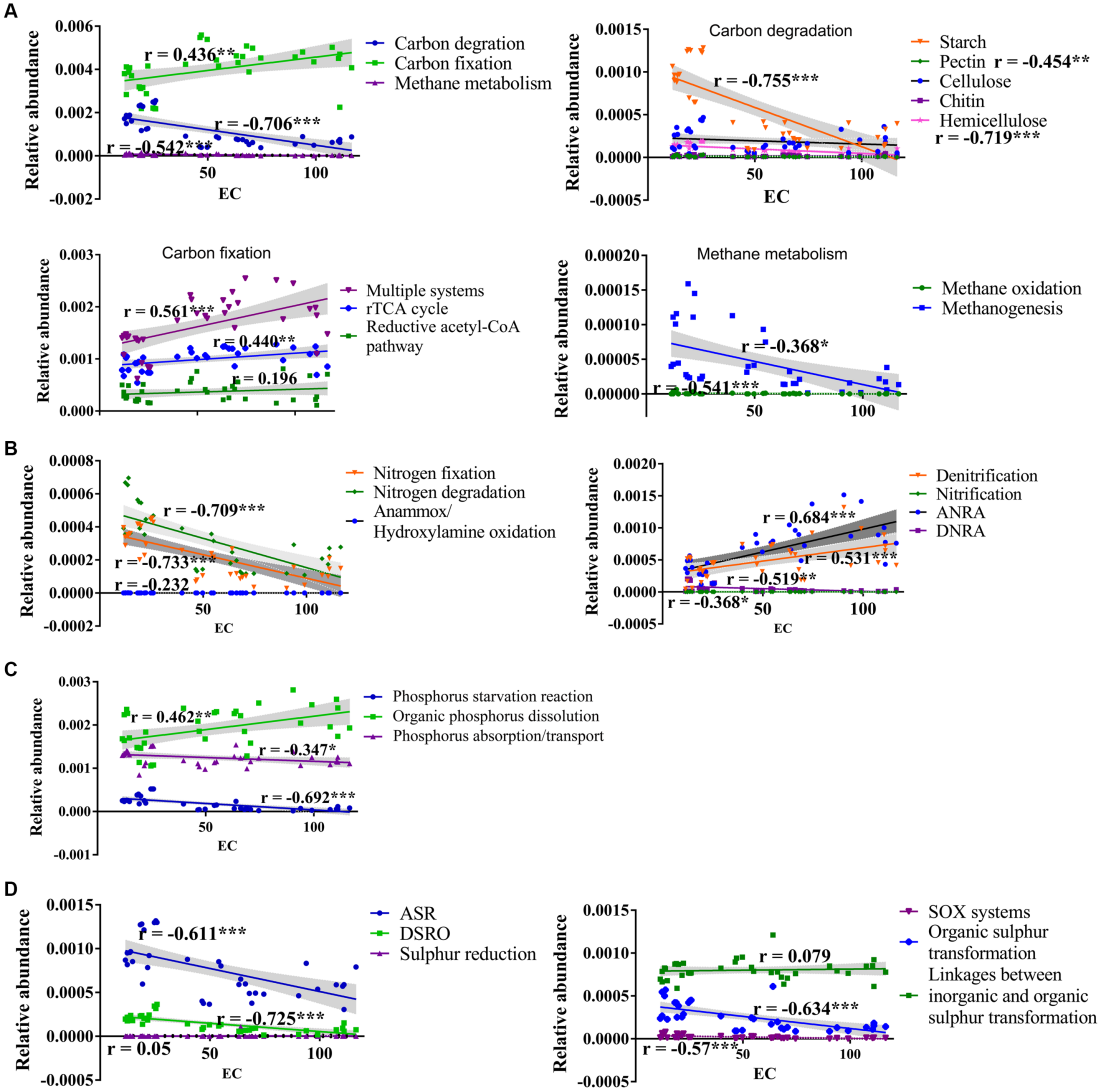
Regression relationships between salt and the main processes participating in the carbon **(A)**, nitrogen **(B)**, phosphorus **(C)**, and sulfur **(D)** cycle.

The carbon cycle mainly consists of carbon fixation, carbon degradation, and methane metabolism, which are crucial pathway for soil microbes to obtain energy and nutrients (Shi et al., 2020). Similar to the previous studies (Yang et al., 2022; Ji et al., 2023), the carbon fixation dominates C cycling function in saline soils of Xinjiang arid region (Figure 2), and salinity enhances carbon fixation, but inhibits carbon degradation pathway (Figure 9A) and genes participating in the degradation of starch, pectin, cellulose, and hemicellulose (Supplementary Figure S2). This could be related to the lower SOC in hypersaline soils compared to that in saline soils. Similarly, salinity has negative effects on soil microbial activity and organic carbon decomposition in arid areas and coastal estuarine wetlands (Yuan et al., 2007; Van Horn et al., 2014; Zhang G. L. et al., 2021; Yang et al., 2023). In contrast, the relative abundance of carbon fixation pathway, such as abundances of the rTCA cycle (and involved genes *korA*, *korB*, *facA*) and the reductive acetyl-CoA pathway (and involved genes *coxL*, *cooC*, *cbbL*) show a positive relationship with soil EC (Figure 9; Supplementary Figure S2). Drought and salt stress suppress the nutrient availability in nutrient, inhibit the growth of microorganism and alter the microbial community composition. The halotolerant or halophilic microbes, i.e., Alphaproteobacteria, Firmicutes, and Euryarchaeota which can adopt rTCA and reductive acetyl-CoA pathway to fix carbon (Fortunato and Huber, 2016; Alfreider et al., 2018; Liu et al., 2018), can survive in the harsh environment. Yang et al. (2022) reported that the halotolerant or halophilic taxa in Alphaproteobacteria, Firmicutes, and Euryarchaeota had higher abundance in hypersaline soils. Therefore, the boost of carbon fixation potential may help microbes growth and be an important mechanism for the maintenance of ecosystem function in in hypersaline soils. Organic carbon degradation typically reflects the consumption characteristics of an ecosystem (Balmonte et al., 2019). A characteristic feature of carbon metabolism in high-salinity environments in desert ecosystems might be that high salinity stabilizes the carbon mechanisms among microbes by reducing carbon degradation and methane metabolism while increasing the carbon fixation process. Simultaneously, less efficient and high-energy-demanding aerobic pathways decrease, whereas highly efficient and low-energy-demanding microaerobic or anaerobic heterotrophic pathways increase, maximizing resource utilization in the carbon cycle process. Besides, salinization inhibits the potential of methane generation evidenced by abundance of methanogenesis and key genes (*mtrA*, *mttB*) negative correlated to EC (Figure 9; Supplementary Figure S2), which is consistent with the findings of other studies showing that the abundance of genes involved in methanogenesis (*mcrA*) decreased with increasing soil salinity (Zhang G. L. et al., 2021; Yang et al., 2023). Previously, Yang et al. reported that the methane metabolism pathway had a weak positive correlation with soil salinity using the whole gene set (Yang et al., 2022).

N elements are essential for microbiomes to withstand environmental pressure (Tyson et al., 2004). In saline habitat, the nitrogen cycling was dominated by denitrification and nitrate assimilation reduction. High salinity significantly inhibits the majority of bacterial nitrogen metabolic function, including nitrogen fixation, nitrification, nitrate assimilation reduction, and nitrogen reduction, consistent with previous findings (Zhang G. L. et al., 2021). The functional genes *nifS*, *nifA*, and *nifH*, involved in nitrogen fixation, are less abundant in hypersaline soils than in saline soils which might be caused by the reduction in the effectiveness of total nitrogen (Supplementary Table S3) which play a crucial role in the growth and metabolism of nitrogen-transforming microbes, and the shifts of microbial community composition (Li X. et al., 2021; Yang et al., 2022). However, salinity prompts denitrification (and genes *narG*, *nirK*, *norB*, and *nosZ*) and assimilation nitrate reduction (ANRA) (and genes *nasA* and *nirA*) (Figure 9; Supplementary Figure S3). Denitrification, where specialized bacteria known as denitrifiers use nitrates or nitrites as alternative electron acceptors in the absence of oxygen, helps to complete the nitrogen cycle by returning nitrogen gas to the atmosphere, where it can be used by plants and other organisms. ANRA is a process by which plants and microorganisms incorporate nitrate (NO_3_^−^) from the environment into organic nitrogen compounds, such as amino acids and proteins, for their own metabolic needs. The study area primarily consists of bare land with extremely low plant coverage, where the efficiency of nitrate usage by plants is very low. The enhancement of these two processes helps regulate the availability of nitrogen in soils, preventing excessive nitrogen accumulation that can lead to environmental problems, and contributes to nitrogen balance in desert saline environments. In addition, the anammox process is important in the removal of nitrogen from various environments. It helps convert ammonium and nitrite into harmless dinitrogen gas. The abundance of *hao* gene primarily found in Proteobacteria and Nitrospirae (Zhang X. Y. et al., 2021), and *amoA* occuring with the archaeal Thaumarchaeota (Bertagnolli and Ulloa, 2017) declined with salinity. This might be caused by the reduction in the abundance of Proteobacteria, Nitrospirae, and Thaumarchaeota from saline to hypersaline environments (Yang et al., 2022).

Salinization increases the abundance of most genes involved in organic phosphate dissolution and organic phosphate mineralization, such as *pstABCS* and *phnCD*, but decreases the abundance of genes involved in phosphate starvation and absorption. The regulation of phosphate starvation by *phoR* and the modulation of phosphate absorption and transport by *gcd* and *ppx* were negatively correlated with the salt gradient (Supplementary Figure S4), in consistent with findings in phosphate-depleted soil or coastal estuarine wetlands (Bergkemper et al., 2016; Zhang G. L. et al., 2021). Salt may enhance phosphate’s effectiveness, at least partially, through the modulation of the phosphate cycle, ultimately enhancing the resupply and unstable phosphate reservoir capacity in salt-affected environments (Hu et al., 2023). The changes in microbial communities and microbial-mediated phosphate cycling may represent strategies for microbial adaptation to high salt stress (Fan et al., 2023; Hu et al., 2023).

The sulfur (S) cycle is an important biogeochemical process in the Earth’s biosphere, as S is a structural component of protein disulfide bonds, amino acids, vitamins, and cofactors. Except for sulfur reduction and linkages between inorganic and organic sulfur transformation, all of the S cycle pathways were negatively correlated with EC (Figure 9D); also, most of the S cycle-related genes’ abundance declined with the EC (Supplementary Figure S5). These findings indicate that salinity significantly inhibits the S cycle. The SOX system plays a critical role in the sulfur oxidation, contributing to the overall sulfur metabolism and energy production. We observes that SOX system is inhibited in hypersaline soil, which is consistent with the findings that sulfur oxidation (*sox*) and sulfite reduction (i.e., *dsr* and *Sir*) decline with the salinity in coastal estuarine wetlands (Zhang G. L. et al., 2021). However, the genes *cysH*, involved in assimilatory sulfate reduction, *cysK*, involved in linkages between inorganic and organic sulfur transformation, and *comS* and *comE*, involved in organic sulfur transformation, have greater abundance in high-salt habitats, revealing the adaptation and response mechanisms of these genes to extreme salt stress in desert environments.

### 4.2 Salinity inhibits the network complexity of element cycles

The complexity and topological characteristics of networks provide a new perspective for microbial composition (In't Zandt et al., 2023). In addition, network analysis can provide information on how microbial communities respond to environmental changes (Hernandez et al., 2021). Interactions among microorganisms shape their functional diversity, and changes in ecological network structure can affect the functionality and stability of ecosystems (Yuan et al., 2021; Jiao et al., 2022). Our findings indicate that high salinization inhibits the complexity of C, N, P, and S genes and environmental networks, and suggest a decrease in interactions among microbial-mediated cycles with increasing salinity, as found in lake microorganisms in arid and semiarid zones (Liu C. Q. et al., 2023).

Positive interactions may reflect cooperation among microbial communities and ecological niche overlap, while negative interactions may indicate competition and niche differentiation among microorganisms (Zhou et al., 2020). In the studied soils, positive interactions dominate all networks, indicating that most microbial species cooperate with one another to establish stable ecological networks. In particular, higher levels of cooperation were present in the nitrogen and sulfur networks in hypersaline soils compared to saline soils. Enhanced network stability suggests that microbial interactions are less susceptible to external disturbances or disruptions, thereby promoting normal growth and even the survival of microorganisms, ultimately contributing to the protection of biodiversity (Czárán et al., 2002; Liu Q. et al., 2023).

Prior research has found that in various cycling processes in saline–alkali environments, the carbon and phosphorus cycles are relatively complex, with microorganisms providing more opportunities for interactions between carbon and phosphorus functions. Compared to simple gene co-occurrence networks, complex networks lead to more efficient resource and information transfer, promoting greater functionality (Li et al., 2023a). Furthermore, highly complex networks result in more stable microbial communities, enhancing tolerance to environmental disturbances. This is because, compared to simple communities, complex microbial communities contain a greater diversity of microbial species, facilitating faster and more complete decomposition of organic matter and phosphorus (Ding et al., 2021). Interestingly, compared to those in saline soils, hypersaline soil microbial community shows an increased proportion of negative links in C and P genes, indicating more competition among microbial communities in high-salt environment.

### 4.3 Driving factors and contributions to community function variation

The effects of edaphic variables on microbial diversity, composition, and activity make up a multifaceted process that involves complex interactions between physical, chemical, and biological factors. Salt is one of the primary influencing factors, which exerts osmotic stress on microorganisms, affecting cell physiology, metabolic activities, and soil biogeochemical processes (Yang et al., 2021; Zhang G. L. et al., 2021). We found that soil salinity primarily has negative effects on microbial functions (Figure 8) along with most of the functional genes involved in these processes (Supplementary Figures S2–S5), also the microbial community network relationships, causing the decline of network complexity, regardless of the element cycle. It is notable that soil salinity enhances functions of denitrification, assimilatory nitrate reduction, and linkages between inorganic and organic sulfur transformation, as well as organic phosphorus dissolution. Soil water content (SWC) is a critical factor influences microbial communities’ structure and function, especially in desert areas, where a low soil water content limits nutrient elements’ dissolution and absorption by plants and microbiomes. We find that soil water content (SWC) negatively affect carbon fixation, denitrification, assimilatory nitrate reduction (ANRA), inorganic P solubilization, organic P mineralization, and P uptake and transport. However, SWC has positive direct impacts on methane metabolism and P starvation response regulation (Figure 8).

Microbes require nutrients for growth, enzyme production, and metabolic functions, including nitrogen, phosphorus, and other essential elements. Nutrient imbalances or deficiencies affect microbial diversity, activity, and ecosystem functioning in saline ecosystems. The desert saline soils were nutrient pools, and salinization further reduced the nutrient availability. The decline in microbial network complexity was significantly correlated with the soil’s SOC, N, and P contents. SEM further supports that AP, ${\mathrm{NH}}_{4}^{+}$ , and ${\mathrm{NO}}_{3}^{-}$ directly promote carbon degradation, denitrification, nitrogen degradation, organic P solubilization and mineralization, P uptake and transport, assimilatory sulfate reduction, and organic sulfur transformation (Figure 8). The availability of P plays a crucial role in stimulating various microbial activities, including the nitrogen cycle (Tang et al., 2016; O'Neill et al., 2022), which is stimulated by the presence of P, as energy synthesis requires elemental P. Ji et al. (2023) found that AP not only enhanced C and S cycling but also contributed to N metabolism, such as nitrogen fixation, nitrification, and denitrification processes, suggesting that P’s availability is essential for maintaining microbial function and improving soil fertility through microbiome manipulation techniques.

This research provides new insights into the effects of soil salinization on nutrient biochemical cycling and in saline environments. Nonetheless, in this study, we only discuss the salinity effects on microbes-mediated C, N, P, and S metabolic functions. As C, N, P, and S cycling are very complex, and our investigation only gain a general insights of how salinity affect nutrient turnover in arid saline soils. Furthermore, the links between the microbial community and functional genes needs investigation to deepen those insights. In the future, field observation and controlling experiments should be conducted to bridge the potential function and actual metabolic activities of microbial communities in the salinized soils.

## 5 Conclusion

This study investigate the response of microbial functional genes involved in key nutrient cycling (carbon, nitrogen, phosphorus, and sulfur) to salinity in arid desert areas. We found that increased the salinity inhibited the carbon degradation, nitrogen fixation, nitrogen degradation, anammox, assimilatory nitrate reduction, dissimilatory nitrate reduction, phosphorus starvation reaction, and phosphorus absorption and transport processes, as well as the majority metabolic pathways in sulfur cycling, whereas enhanced the carbon fixation, denitrification, ANRA, and organic phosphorus dissolution. This suggests that salinity affects changes in microbial functional genes related to biogeochemical nutrient turnover. High salinization inhibits the complexity of C, N, P, and S genes and environmental networks. Our results also showed that with the increase of salt stress, there was a higher level of cooperation between nitrogen and sulfur networks, and a higher proportion of negative links in C and P genes. This indicates that the relationship between different microbial community may varies in response to salt stress. Soil salinity was the most important factor driving microbial functions, beside, nutrient availability also played an important role in microbiomes functions and interactions; the most important were AP, ${\mathrm{NH}}_{4}^{+}$ , and ${\mathrm{NO}}_{3}^{-}$ , which directly promoted carbon fixation, denitrification, organic P solubilization, P uptake and transport, assimilatory sulfate reduction, and organic sulfur transformation.

## Data availability statement

The datasets presented in this study can be found in online repositories. The names of the repository/repositories and accession number(s) can be found in the article/Supplementary material.

## Author contributions

YL: Conceptualization, Writing – original draft. WL: Formal analysis, Visualization, Writing – review & editing. LJ: Formal analysis, Writing – review & editing. EL: Formal analysis, Writing – review & editing. XY: Data curation, Investigation, Writing – review & editing. JY: Conceptualization, Funding acquisition, Supervision, Writing – review & editing.
