# Supplementary material for: Salinity affects microbial function genes related to nutrient cycling in arid regions
Source: Front Microbiol. 2024 Jun 14;15:1407760. doi: 10.3389/fmicb.2024.1407760 (PMC11212614; doi:10.3389/fmicb.2024.1407760)
Supplement: Supplementary file 1 [file Data_Sheet_1.docx]

Supplementary Material

## Supplementary Figures


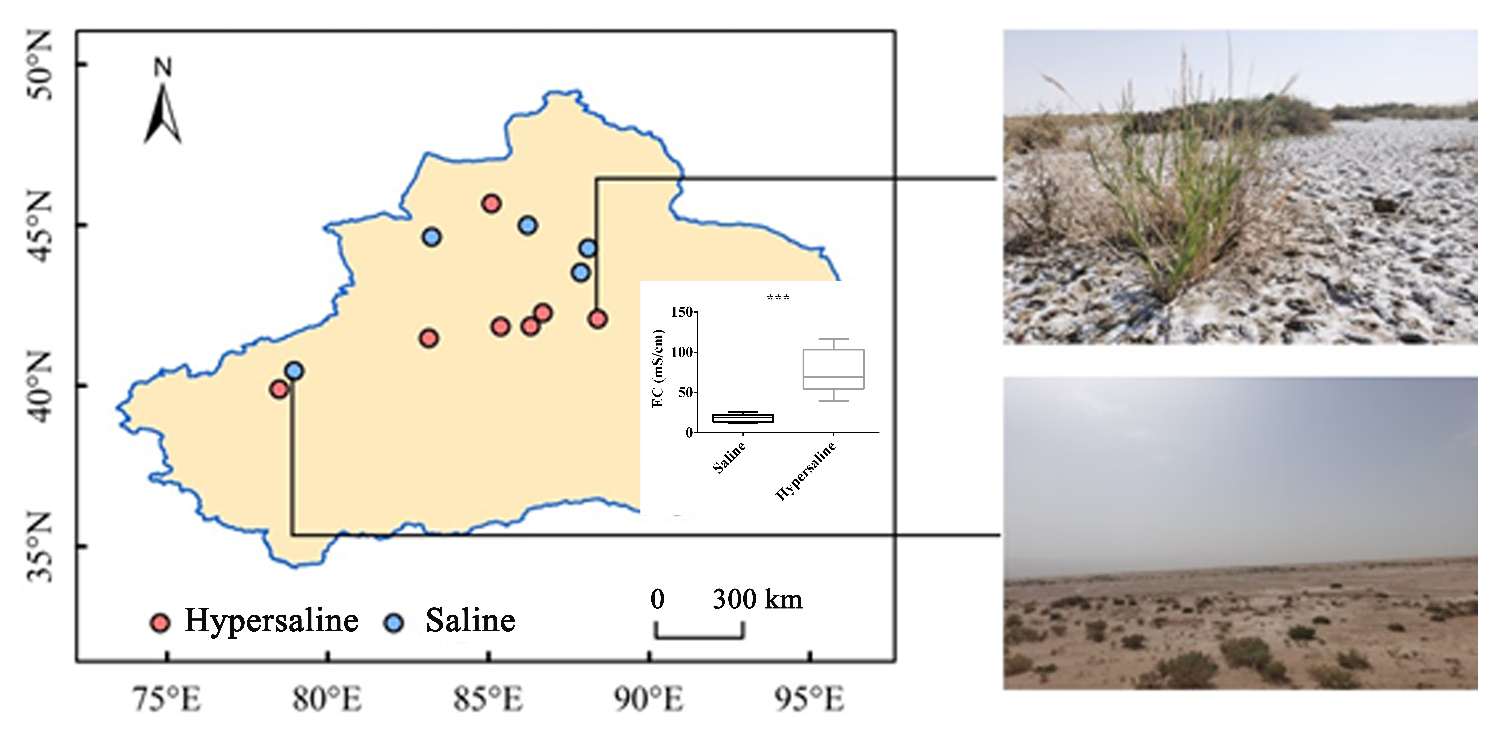


Supplementary FIGURE 1. Soil physicochemical properties at each sampling site; Figure S1. Illustration of sampling locations of saline soils collected from Xinjiang, China. Saline represents soils with EC < 30, with a mean value of 18.32 ± 1.294 (N = 15), while hypersaline represents soils with EC > 30, with a mean value 76.58 ± 5.464 (N=21). There are significant differences in salinity between the saline and hypersaline soils (*p* < 0.001);





Supplementary FIGURE 2. Linear regression between key genes involved in carbon cycling processes and salinity (EC value);





Supplementary FIGURE 3. Linear regression between key genes involved in nitrogen cycling processes and salinity (EC value);





Supplementary FIGURE 4. Linear regression between key genes involved in phosphorus cycling processes and salinity (EC value);





Supplementary FIGURE 5. Linear regression between key genes involved in sulfur cycling processes and salinity (EC value).

## Supplementary Tables

Supplementary TABLE 1 The geographic information and soil physicochemical properties in sampling location

| Name | Location | Coordinate | AP  (mg kg^−1^) | EC  (mS cm^−1^) | pH | SOC  (g kg^−1^) | TN  (g kg^−1^) | TP  (g kg^−1^) | 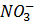^—^N  (mg kg^−1^) | 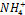-N  (mg kg^−1^) | SWC  (%) |
| --- | --- | --- | --- | --- | --- | --- | --- | --- | --- | --- | --- |
| ZNQ | Yanqi, Xinjiang | 86°19′E, 41°50′N | 13.40±0.62 | 64.47±0.90 | 8.16±0.07 | 9.25±0.24 | 0.49±0.01 | 0.48±0.01 | 156.6±1.98 | 1.12±0.01 | 5.59±0.43 |
| KMS | Tuokexun, Xinjiang | 88°24′E, 42°05′N | 10.51±1.49 | 88.03±7.17 | 7.74±0.05 | 7.14±0.52 | 0.37±0.01 | 0.70±0.01 | 100.90±3.74 | 0.82±0.10 | 2.81±0.32 |
| KCB | Kuche, Xinjiang | 83°09′E, 41°28′N | 3.55±0.25 | 106.00±6.60 | 7.93±0.05 | 6.14±0.64 | 0.24±0.00 | 0.46±0.01 | 32.62±0.76 | 0.66±0.03 | 5.65±0.40 |
| TMG | Timenguan, Xinjiang | 85°23′E, 41°50′N | 3.83±0.12 | 47.73±1.05 | 7.88±0.05 | 1.61±0.26 | 0.20±0.00 | 0.52±0.01 | 162.8±4.46 | 0.45±0.08 | 10.89±0.26 |
| HSB | Heshuo, Xinjiang | 86°42′E, 42°16′N | 23.87±0.38 | 110.90±0.20 | 8.70±0.01 | 12.68±0.07 | 0.55±0.00 | 0.37±0.00 | 242.4±6.36 | 1.74±0.05 | 9.10±0.47 |
| BJT | Kelamayi, Xinjiang | 85°6′E, 45°40′N | 5.58±0.38 | 49.60±4.91 | 8.39±0.07 | 6.93±0.19 | 0.28±0.01 | 0.44±0.01 | 115.4±4.38 | 2.24±0.15 | 5.89±0.94 |
| BCB | Baicheng, Xinjiang | 78°30′E, 39°53′N | 2.60±0.81 | 69.27±0.81 | 7.59±0.01 | 3.28±0.17 | 0.39±0.01 | 0.41±0.01 | 208±3.32 | 1.45±0.09 | 10.31±1.17 |
| ABH | Jinghe, Xinjiang | 83°14′E, 44°37′N | 4.17±0.22 | 21.08±0.90 | 8.22±0.02 | 1.43±0.14 | 0.18±0.00 | 0.27±0.00 | 5.64±0.28 | 0.49±0.06 | 11.99±0.40 |
| KPB | Keping, Xinjiang | 78°58′E, 40°27′N | 4.97±0.97 | 12.87±0.97 | 7.87±0.01 | 2.49±0.05 | 0.30±0.01 | 0.47±0.01 | 143.10±3.46 | 1.10±0.05 | 4.54±0.53 |
| YSJ | Manasi, Xinjiang | 86°14′E, 44°59′N | 11.22±0.34 | 19.07±0.48 | 8.33±0.01 | 2.16±0.05 | 0.33±0.00 | 0.49±0.00 | 120.20±2.61 | 1.32±0.06 | 11.99±0.40 |
| FKC | Fukang, Xinjiang | 88°06′E, 44°16′N | 25.44±0.72 | 25.33±0.35 | 8.62±0.03 | 4.14±0.37 | 0.33±0.01 | 0.39±0.01 | 16.01±1.67 | 0.91±0.05 | 4.54±0.53 |
| CWP | Urumqi, Xinjiang | 87°52′E, 43°31′N | 45.52±3.55 | 13.27±0.45 | 9.76±0.02 | 25.28±0.07 | 2.46±0.05 | 0.64±0.02 | 531.00±6.93 | 2.53±0.18 | 8.60±0.18 |
| Total Mean | / | / | 12.89±2.10 | 52.30±5.81 | 8.27±0.09 | 6.88±1.09 | 0.51±0.10 | 0.47±0.02 | 152.91±22.61 | 1.24±0.11 | 6.99±0.55 |
| Hypersaline | / | / | 9.05±1.60b | 76.58±5.46a | 8.05±0.08a | 6.72±0.77a | 0.36±0.03b | 0.48±0.02a | 145.57±14.52b | 1.21±0.13a | 7.17±0.65a |
| Saline | / | / | 18.26±4.22a | 18.32±1.29b | 8.56±0.17a | 7.10±2.44b | 0.72±0.23a | 0.45±0.03a | 163.19±51.30a | 1.27±0.19a | 6.72±0.99a |

Values in Mean ± Std. Deviation. SWC, soil water content; SOC, soil organic carbon content; TN, soil total nitrogen content; TP, soil total phosphorous content; AP, soil available phosphorous content; NO_3_^-^-N, soil nitrate nitrogen; NH_4_^+^-N, soil ammonium nitrogen. Different lowercase letters represent significant differences between saline and hypersaline soils at *p* < 0.05.

Supplementary TABLE 2 Functional genes surveyed in present study

| Cycle | Process | item | KO id | Gene |
| --- | --- | --- | --- | --- |
| Carbon cycle | Carbon degradation | Starch | K00705 | *malQ* |
|  |  |  | K01187 | *malZ* |
|  |  |  | K01214 | *iam* |
|  |  | Pectin | K01728 | *pel* |
|  |  |  | K01184 | *pgl* |
|  |  | Cellulose | K01125 | *cbh* |
|  |  |  | K19668 | *cbhA* |
|  |  |  | K01179 | *celF* |
|  |  | Chitin | K01183 | *chi* |
|  |  | Hemicellulose | K01710 | *rfbB* |
|  |  |  | K01805 | *xylA* |
|  |  |  | K10543 | *xylF* |
|  |  |  | K10544 | *xylH* |
|  | Carbon fixation | Multiple systems | K01962 | *accA* |
|  |  |  | K01895 | *facA* |
|  |  |  | K11263 | *pccA* |
|  |  | Reductive tricarboxylic acid cycle (rTCA cycle) | K00031 | *icd* |
|  |  |  | K00174 | *korA* |
|  |  |  | K00175 | *korB* |
|  |  | Reductive acetyl-CoA pathway | K07321 | *cooC* |
|  |  |  | K01938 | *fhs* |
|  |  |  | K01601 | *cbbL* |
|  |  |  | K21703 | *cbbR* |
|  |  |  | K01602 | *cbbS* |
|  |  |  | K03520 | *coxL* |
|  |  |  | K03519 | *coxM* |
|  |  |  | K03518 | *coxS* |
|  | Methane metabolism | Methanogenesis | K00577 | *mtrA* |
|  |  |  | K14083 | *mttB* |
| Nitrogen cycle | Nitrogen fixation |  | K02584 | *nifA* |
|  |  |  | K04487 | *nifS* |
|  |  |  | K02588 | *nifH* |
|  |  |  | K02586 | *nifD* |
|  |  |  | K02591 | *nifK* |
|  | Denitrification |  | K00370 | *narG* |
|  |  |  | K00371 | *narH* |
|  |  |  | K00368 | *nirK* |
|  |  |  | K15864 | *nirS* |
|  |  |  | K04561 | *norB* |
|  |  |  | K02305 | *norC* |
|  |  |  | K00376 | *nosZ* |
|  | Nitrification |  | K10944 | *amoA/pmoA* |
|  |  |  | K10945 | *amoB/pmoB* |
|  |  |  | K10946 | *amoC/pmoC* |
|  | Assimilatory nitrate reduction (ANRA) |  | K00372 | *nasA* |
|  |  |  | K15578 | *nasD* |
|  |  |  | K00366 | *nirA* |
|  | Dissimilatory nitrate reduction (DNRA) |  | K02567 | *napA* |
|  |  |  | K02568 | *napB* |
|  |  |  | K02569 | *napC* |
|  |  |  | K03385 | *nrfA* |
|  |  |  | K15876 | *nrfH* |
|  | Anammox/Hydroxylamine oxidation |  | K10535 | *hao/hzo* |
|  | Nitrogen degradation |  | K01428 | *ureC* |
|  |  |  | K00262 | *GDH1* |
|  |  |  | K15371 | *GDH2* |
| Phosphorus cycle | Phosphorus starvation reaction |  | K07636 | *phoR* |
|  |  |  | K07657 | *phoB* |
|  | Organic phosphorus dissolution |  | K05813 | *ugpB* |
|  |  |  | K05814 | *ugpA* |
|  |  |  | K05815 | *ugpE* |
|  |  |  | K05816 | *ugpC* |
|  |  |  | K02036 | *pstB* |
|  |  |  | K02037 | *pstC* |
|  |  |  | K02038 | *pstA* |
|  |  |  | K02040 | *pstS* |
|  |  |  | K02041 | *phnC* |
|  |  |  | K02042 | *phnE* |
|  |  |  | K02044 | *phnD* |
|  | Phosphorus absorption/transport |  | K00117 | *gcd* |
|  |  |  | K01077 | *phoA* |
|  |  |  | K01078 | *olpA* |
|  |  |  | K01093 | *appA* |
|  |  |  | K01113 | *phoD* |
|  |  |  | K01126 | *ugpQ* |
|  |  |  | K01507 | *ppa* |
|  |  |  | K01524 | *ppx* |
|  |  |  | K03430 | *phnW* |
|  |  |  | K05306 | *phnX* |
|  |  |  | K05774 | *phnN* |
|  |  |  | K05780 | *phnL* |
|  |  |  | K06162 | *phnM* |
|  |  |  | K06163 | *phnJ* |
|  |  |  | K06164 | *phnI* |
|  |  |  | K06165 | *phnH* |
|  |  |  | K06166 | *phnG* |
|  |  |  | K06167 | *phnP* |
|  |  |  | K09474 | *phoN* |
| Sulfur cycle | Assimilatory sulphate reduction |  | K00860 | *cysC* |
|  |  |  | K00957 | *cysND* |
|  |  |  | K00390 | *cysH* |
|  |  |  | K00380 | *cysI* |
|  |  |  | K00381 | *cysJ* |
|  |  |  | K00955 | *cysNC* |
|  |  |  | K00956 | *cysN* |
|  |  |  | K00392 | *sir* |
|  | Dissimilatory sulphur reduction and oxidation |  | K07648 | *aprAB1* |
|  |  |  | K07773 | *aprAB2* |
|  |  |  | K00394 | *aprA* |
|  |  |  | K11181 | *dsrB* |
|  |  |  | K08738 | *CYC* |
|  |  |  | K00958 | *sat* |
|  | Sulphur reduction |  | K17216 | *mccA* |
|  | SOX system |  | K17222 | *soxA* |
|  |  |  | K17223 | *soxX* |
|  |  |  | K17224 | *soxB* |
|  |  |  | K17225 | *soxCD* |
|  |  |  | K17226 | *soxY* |
|  |  |  | K17227 | *soxZ* |
|  | Organic sulphur transformation |  | K00108 | *betA* |
|  |  |  | K00130 | *betB* |
|  |  |  | K08097 | *comA* |
|  |  |  | K05979 | *comB* |
|  |  |  | K05884 | *comS* |
|  |  |  | K06034 | *comCD* |
|  |  |  | K13039 | *comE* |
|  |  |  | K13788 | *pta* |
|  | Linkages between inorganic and organic sulphur transformation |  | K00640 | *cysE* |
|  |  |  | K01738 | *cysK* |
|  |  |  | K12339 | *cysM* |
|  |  |  | K03388 | *hdrA* |
|  |  |  | K03389 | *hdrB* |
|  |  |  | K03390 | *hdrC* |
|  |  |  | K17217 | *mccB* |
|  |  |  | K00651 | *metA* |
|  |  |  | K01739 | *metB* |
|  |  |  | K01760 | *metC* |
|  |  |  | K15760 | *tmoA* |
|  |  |  | K15761 | *tmoB* |
|  |  |  | K15762 | *tmoC* |
|  |  |  | K15764 | *tmoE* |
|  |  |  | K15765 | *tmoF* |
|  | Other |  | K02045 | *cysA* |
|  |  |  | K02048 | *cysP* |
|  |  |  | K02047 | *cysW* |
|  |  |  | K15553 | *ssuA* |
|  |  |  | K15555 | *ssuB* |
|  |  |  | K15554 | *ssuC* |
|  |  |  | K15551 | *tauA* |
|  |  |  | K10831 | *tauB* |
|  |  |  | K15552 | *tauC* |
|  | | | | |

Supplementary TABLE 3 The correlation between soil variables

|  | EC | AP | pH | SOC | TN | TP | NO3 | NO_3_^-^ | SWC |
| --- | --- | --- | --- | --- | --- | --- | --- | --- | --- |
| EC | 1 |  |  |  |  |  |  |  |  |
| AP | -0.203 | 1 |  |  |  |  |  |  |  |
| pH | -0.331^*^ | **0.902**^**^ | 1 |  |  |  |  |  |  |
| SOC | 0.065 | **0.843**^**^ | **0.807**^**^ | 1 |  |  |  |  |  |
| TN | -0.26 | **0.847**^**^ | **0.820**^**^ | **0.915**^**^ | 1 |  |  |  |  |
| TP | 0.036 | 0.306 | 0.115 | 0.429^**^ | 0.463^**^ | 1 |  |  |  |
| NO_3_^-^ | -0.127 | 0.696^**^ | 0.651^**^ | **0.838**^**^ | 0.898^**^ | 0.460^**^ | 1 |  |  |
| NH_4_^+^ | -0.105 | 0.577^**^ | 0.647^**^ | 0.708^**^ | 0.667^**^ | 0.190 | 0.720^**^ | 1 |  |
| SWC | -0.019 | 0.047 | 0.13 | 0.06 | 0.115 | -.457^**^ | 0.169 | -0.086 | 1 |

Note: *, ** and *** represent *p* < 0.05, *p* < 0.01, and *p* < 0 .001, respectively.
